# A Functional Variant in *SEPP1* Interacts With Plasma Selenium Concentrations on 3-Year Lipid Changes: A Prospective Cohort Study

**DOI:** 10.3389/fnut.2021.789577

**Published:** 2021-12-07

**Authors:** Li Zhou, Xiaoling Liang, Manling Xie, Jiawei Yin, Yue Huang, Xiaoqin Li, Zhilei Shan, Liangkai Chen, Yan Zhang, Cheng Luo, Liegang Liu

**Affiliations:** ^1^Department of Nutrition and Food Hygiene, Hubei Key Laboratory of Food Nutrition and Safety, School of Public Health, Tongji Medical College, Huazhong University of Science and Technology, Wuhan, China; ^2^Ministry of Education (MOE) Key Lab of Environment and Health, School of Public Health, Tongji Medical College, Huazhong University of Science and Technology, Wuhan, China; ^3^Academy of Nutrition and Health, Wuhan University of Science and Technology, Wuhan, China; ^4^Department of Neurology, Mayo Clinic, Rochester, MN, United States; ^5^The Hubei Provincial Key Laboratory of Yeast Function, Yichang, China

**Keywords:** selenium, *SEPP1*, gene-environment interactions, lipid changes, cohort study

## Abstract

**Background:** Excess selenium has been related with adverse lipid levels in previous epidemiological studies. Meanwhile, a functional variant in *SEPP1* (encodes selenoprotein P), namely rs7579, has been suggested to modulate lipid metabolism. However, the interactions between selenium status and rs7579 polymorphism on lipid changes remain unclear.

**Objective:** To examine whether the associations between plasma selenium and 3-year lipid changes is modified by rs7579 polymorphism.

**Methods:** A prospective cohort study was conducted among 1,621 individuals to examine the associations between baseline plasma selenium and 3-year lipid changes, as well as the interactions between plasma selenium and rs7579 polymorphism on lipid changes.

**Results:** The median (interquartile range) concentration of plasma selenium was 91.68 (81.55–104.92) μg/L. Higher plasma selenium was associated with adverse 3-year lipid changes. Comparing the highest to the lowest quartiles of plasma selenium concentrations, 3-year lipid changes were elevated by 8.25% (95% CI: 1.54–14.96%) for triglycerides (*P* = 0.016), 5.88% (3.13–8.63%) for total cholesterol (*P* < 0.001), 7.37% (3.07–11.67%) for low-density lipoprotein cholesterol (*P* = 0.0008), 6.44% (2.66–10.21%) for non-high-density lipoprotein cholesterol (*P* = 0.0009), 4.99% (0.62–9.36%) for total cholesterol/high-density lipoprotein cholesterol ratio (*P* = 0.025), and 7.00% (1.55–12.46%) for low-density lipoprotein cholesterol/high-density lipoprotein cholesterol ratio (*P* = 0.012). In analyses stratified by rs7579 genotypes, positive associations between plasma selenium concentrations and 3-year changes in triglycerides, TC, LDL-C, non-HDL-C, TC/HDL-C ratio, and LDL-C/HDL-C ratio were observed among CC genotype carriers, but negative associations between plasma selenium and TC/HDL-C ratio, and LDL-C/HDL-C ratio were observed among TT genotype carriers.

**Conclusions:** Our findings suggested that plasma selenium was associated with 3-year lipid changes differentially by rs7579 genotypes, and higher plasma selenium was associated with adverse lipid changes among rs7579 CC genotype carriers, but not among T allele carriers.

## Introduction

Selenium is an essential trace element which plays an important role in the antioxidant process by incorporating into selenoproteins (1). Because of this property, selenium supplementation has been suggested to protect against oxidative stress. In recent years, selenium-containing supplements and selenium-rich foods have been widely used due to their potential benefits (2). However, several studies revealed that high selenium was associated with numerous metabolic disorders, including dyslipidemia (3–5). Most epidemiological studies examining the associations between selenium status and lipid levels are cross-sectional, while longitudinal evidence to show prospective associations is limited (3, 6–8).

Genetic factors are believed to play an important role in lipid metabolism (9, 10). A functional variant located in the 3′ untranslated regions of *SEPP1*, namely rs7579 polymorphism, has been suggested to modulate lipid response to Brazil nut supplementation, and Brazil nut is the richest natural source of selenium (11). *SEPP1* encodes selenoprotein P (SELENOP), which mainly functions as a selenium transporter and delivers selenium from the liver to other tissues (12, 13). Moreover, findings from previous studies indicated that SELENOP played a crucial role in the antioxidant-mediated protection against colitis-associated cancer (14, 15). Several studies demonstrated that the rs7579 polymorphism affected the mRNA expression as well as plasma levels of SELENOP, and both the mRNA expression and plasma levels of SELENOP were significantly lower among rs7579 CC genotype than T allele carriers (16, 17). Meanwhile, previous findings showed that the changes in cholesterol levels were greater among rs7579 CC genotype than T allele carriers in response to Brazil nut supplementation (18). Based on the function of rs7579 polymorphism, it can be anticipated that the associations between plasma selenium and changes in lipid levels are more pronounced in rs7579 CC genotype than T allele carriers. However, to our knowledge, no study has examined the interaction between plasma selenium and rs7579 polymorphism on lipid changes.

Hence, the current study aimed to evaluate the associations between plasma selenium concentrations and changes in lipid levels, and then to examine whether the associations differ across rs7579 genotypes.

## Methods

### Study Design and Subjects

The current study was conducted based on the Tongji-Ezhou (TJEZ) cohort study, which is an ongoing prospective cohort study, with the aim of investigating the associations of lifestyle, dietary factors, and genetic factors with chronic diseases. Design of the TJEZ cohort study has been described elsewhere (5). Briefly, 5,533 residents in Ezhou aged above 20 years were recruited between 2013 and 2015. At baseline, all participants underwent comprehensive medical examinations, including semi-structured questionnaires, anthropometric measurements, and biochemical analyses (including lipid measurements). Also, fasting venous blood samples were collected from all participants at baseline enrollment. All blood samples were separated for plasma within 1 h and stored in −80°C freezers until laboratory analysis. Additionally, genotyping of rs7579 was performed on 3,605 subjects at baseline enrollment. The TJEZ cohort will be followed up every 3 years, and a total of 4,695 subjects participated in the first follow-up survey which was conducted between 2016 and 2018, with a follow-up rate of 91.1%. In the first follow-up survey, 3,204 cohort members were invited to undergo lipid remeasurements and genotyping; the 2,691 (84.0%) respondents were broadly representative of the total cohort; 1,957 subjects had lipid measurements, and 1,655 individuals among them had genotyping results at baseline. After excluding subjects on lipid-lowering drugs, a subset of 1,621 subjects who had genotyping results at baseline as well as lipid measurements in the first follow-up survey, were included in the present study.

The study was approved by the Ethics and Human Subject Committee of Tongji Medical College, and written informed consent was obtained from all participants.

### Lipid Measurement

Fasting serum lipid levels, including triglycerides, total cholesterol (TC), high-density lipoprotein cholesterol (HDL-C), low-density lipoprotein cholesterol (LDL-C), were measured by Hitachi automatic analyzer. Both the intra-assay and inter-assay variation coefficients of lipid levels were below 5%. The non-high-density lipoprotein cholesterol (non-HDL-C) level was calculated as TC subtracting HDL-C. Additionally, the TC/HDL-C and LDL-C/HDL-C ratio were calculated. Changes in lipid levels during 3-year follow-up period were calculated by subtracting the lipids at follow-up from lipids at baseline.

### Measurement of Plasma Selenium Concentrations

Plasma selenium concentrations were measured by inductively coupled plasma mass spectrometry (Agilent 7700 Series, Tokyo, Japan) in the Ministry of Education Key Laboratory of Environment and Health at Tongji Medical College of Huazhong University of Science & Technology, as described previously (5). Prior to analysis, thawed samples (40 μL plasma) were diluted with ultrapure water and digestive solution at a ratio of 1:19:20. For quality assurance, standard reference material ClinChek No. 8883 and No. 8884 human plasma controls for trace elements were analyzed in every 20 samples. The detection limit for plasma selenium was 0.024 μg/L, and no samples had a level below this limit. Both intra-assay and inter-assay variation coefficients of plasma selenium were below 5%.

### Genotyping

Genomic DNA was isolated from the peripheral blood sample by kit (Tiangen biotech, Beijing, China). The sample DNA was amplified by a multiplex polymerase chain reaction for locus-specific single-base extension reaction. Genotyping of rs7579 was done by the MassArray system (Agena iPLEXassay, San Diego, United States). The sequences for the forward primer are “ACGTTGGATGTGACGCTGAAAGAATCAGGC,” and the sequences for the reverse primer are “ACGTTGGATGTGTGTCTAGACTAAATTGGG.” Genotyping was successful in 99.3% (1,609 of 1,621) of the participants. The genotypes distribution of rs7579 was in Hardy-Weinberg equilibrium.

### Assessment of Covariates

Basic information including sex, age, smoking status, alcohol drinking status, physical activity, educational level, and medical history (including use of lipid-lowering medication) was obtained *via* semi-structured questionnaires at baseline and was included as adjustment variables. Smoking status was grouped into two categories: current smoking (≥1 cigarette/day over the past 6 months) and non-smoking. Alcohol drinking status was classified as current drinking (≥1 time/week over the past 6 months) and non-drinking. Physical activity was defined as regular exercise for at least 60 min per week over the past 6 months. We classified educational level into three categories: none or elementary school, middle school, and high school or college. Anthropometric measurements were performed by trained staff. Body weight and standing height were measured in light indoor clothing and without shoes. Body mass index (BMI) was calculated by dividing the weight in kilograms by the square of the height in meters.

### Statistical Analysis

Descriptive statistics were shown as means ± standard deviations for continuous variables if normally distributed, medians (interquartile ranges) for continuous variables if not normally distributed, and frequencies (percentages) for categorical variables. Basic characteristics were compared across quartiles of plasma selenium by analysis of variance (normal distribution) or Kruskal-Wallis test (skewed distribution) for continuous variables, and χ^2^-test for categorical variables. Multivariable linear regression models were used and regression coefficients (95% CIs) were calculated to examine the associations of plasma selenium concentrations, as well as rs7579 polymorphisms, with 3-year changes in lipid levels. In multivariate models, we adjusted for respective baseline lipid (continuous) in model 1. In model 2, we further adjusted for sex (men/women), age (continuous), and BMI (continuous). In model 3, we further adjusted for current smoking status (yes/no), current drinking status (yes/no), physical activity (yes/no), and educational level (none or elementary school/middle school/high school or beyond). Due to right-skewed distribution, all lipids were ln-transformed and multiplied by 100 before analyses. Changes in lipid levels during 3-year follow-up period were calculated by subtracting the 100^*^ln-transformed lipids at follow-up from 100^*^ln-transformed lipids at baseline. Hence, regression coefficients in models can be interpreted as percentage difference in means (19).

To examine whether the associations between plasma selenium and 3-year changes in lipid levels were different across rs7579 genotypes, analyses of plasma selenium with 3-year lipid changes were stratified by rs7579 genotypes, and interactions between plasma selenium and rs7579 on 3-year lipid changes were identified through 1df multiplicative terms tests. Statistical power for the selenium × rs7579 interactions with lipid changes was calculated using QUANTO 1.2.4 (http://biostats.usc.edu/Quanto.html). Assuming a marginal $R_{G}^{2}$ of 0.008, a marginal $R_{E}^{2}$ of 0.01, and a marginal $R_{GE}^{2}$ of 0.006, our study had 88% power to detect an interaction for a lipid change.

All analyses were conducted using SAS 9.4 (SAS Instituted Inc, Cary, NC), and two-side *P* < 0.05 were considered statistically significant.

## Results

General characteristics of participants are shown in Table 1. The 1,621 participants had a mean age of 57.86 years, and 65.3% were male. The median (interquartile range) concentration of plasma selenium was 91.68 (81.55–104.92) μg/L. At baseline, participants with higher plasma selenium concentrations were more likely to be men, current drinkers, and had higher levels of TC, LDL-C, and non-HDL-C.

**Table 1:** Baseline characteristics of participants^a^.

**Characteristics**	**Total**	**Quartiles of plasma selenium concentrations (μg/L)**				***P*-value**
		**Q1, <81.56**	**Q2, 81.57–91.68**	**Q3, 91.69–104.90**	**Q4, ≥104.91**	
No of participants	1,621	406	405	405	405	
Men, *n* (%)	1,058 (65.3)	253 (62.3)	256 (63.2)	257 (63.5)	292 (72.1)	**0.010**
Age, years	57.86 (10.25)	56.97 (11.20)	58.16 (10.72)	58.13 (9.54)	58.19 (9.40)	0.247
BMI, kg/m^2^	23.79 (3.13)	23.80 (3.27)	23.72 (3.02)	23.61 (3.15)	24.03 (3.06)	0.273
Triglycerides, mmol/L	1.24 (0.88, 1.78)	1.19 (0.84, 1.74)	1.28 (0.92, 1.84)	1.23 (0.89, 1.70)	1.28 (0.89, 1.96)	0.105
TC, mmol/L	4.75 (4.18, 5.36)	4.58 (3.91, 5.12)	4.62 (4.10, 5.31)	4.87 (4.28, 5.43)	4.95 (4.32, 5.56)	**<0.001**
HDL-C, mmol/L	1.32 (1.10, 1.58)	1.27 (1.05, 1.54)	1.33 (1.09, 1.57)	1.35 (1.14, 1.59)	1.34 (1.11, 1.61)	**0.008**
LDL-C, mmol/L	2.82 (2.28, 3.33)	2.72 (2.15, 3.17)	2.79 (2.25, 3.31)	2.87 (2.41, 3.36)	2.89 (2.29, 3.56)	**<0.001**
Non-HDL-C, mmol/L	3.37 (2.80, 4.01)	3.20 (2.65, 3.78)	3.29 (2.80, 3.99)	3.48 (2.86, 4.06)	3.58 (2.98, 4.20)	**<0.001**
TC/HDL-C ratio	3.56 (2.96, 4.36)	3.51 (2.87, 4.37)	3.57 (2.94, 4.30)	3.51 (2.94, 4.29)	3.66 (3.05, 4.49)	0.154
LDL-C/HDL-C ratio	2.12 (1.63, 2.72)	2.10 (1.63, 2.72)	2.11 (1.64, 2.72)	2.08 (1.59, 2.71)	2.21 (1.71, 2.75)	0.549
Education level, *n* (%)						0.091
None or elementary school	424 (26.2)	99 (24.4)	95 (23.5)	105 (25.9)	125 (30.9)	
Middle school	734 (45.3)	174 (42.8)	196 (48.4)	191 (47.2)	173 (42.7)	
High school or beyond	463 (28.5)	133 (32.8)	114 (28.1)	109 (26.9)	107 (26.4)	
Current smoker, *n* (%)	479 (29.5)	120 (29.6)	122 (30.1)	116 (28.6)	121 (29.9)	0.970
Current drinker, *n* (%)	480 (29.6)	99 (24.4)	109 (26.9)	120 (29.6)	152 (37.5)	**<0.001**
Vigorous activity, *n* (%)	738 (45.5)	180 (44.3)	198 (48.9)	177 (43.7)	183 (45.2)	0.450
rs7579 genotypes, *n* (%)^b^						
CC	882 (54.7)	209 (51.7)	235 (58.1)	225 (56.1)	213 (53.0)	0.234
CT	614 (38.1)	159 (39.4)	140 (34.7)	156 (38.9)	159 (39.5)	
TT	115 (7.1)	36 (8.9)	29 (7.2)	20 (5.0)	30 (7.5)	
Plasma selenium, μg/L	91.68 (81.55, 104.92)	75.25 (68.92, 78.50)	87.09 (84.16, 89.11)	97.86 (94.50, 101.08)	114.67 (108.91, 126.16)	**<0.001**

*^a^Data are presented as mean (SD) for parametrically distributed data, median (interquartile range) for non-parametrically distributed data, or n (%) for categorical data. BMI, body mass index; HDL-C, high-density lipoprotein cholesterol; LDL-C, low-density lipoprotein cholesterol; Non-HDL-C, non-high-density lipoprotein cholesterol; TC, total cholesterol.*

*^b^Genotyping of rs7579 polymorphism was failed in 12 participants. Bold values indicates P < 0.05*.

After adjustment for sex, age, BMI, educational level, lifestyle factors, and respective baseline lipid, plasma selenium concentrations were positively associated with 3-year changes in triglycerides, TC, LDL-C, non-HDL-C, TC/HDL-C ratio, and LDL-C/HDL-C ratio (Table 2). Comparing the highest to the lowest quartiles of plasma selenium concentrations, 3-year changes in lipid levels were elevated by 8.25% (95% CI: 1.54–14.96%) for triglycerides (*P* = 0.016), 5.88% (3.13–8.63%) for TC (*P* < 0.001), 7.37% (3.07–11.67%) for LDL-C (*P* = 0.0008), 6.44% (2.66–10.21%) for non-HDL-C (*P* = 0.0009), 4.99% (0.62–9.36%) for TC/HDL-C ratio (*P* = 0.025), and 7.00% (1.55–12.46%) for LDL-C/HDL-C ratio (*P* = 0.012). The associations between rs7579 polymorphism and 3-year changes in lipids were showed in Table 3. After adjustment for sex, age, BMI, educational level, lifestyle factors, and respective baseline lipid, 3-year changes in lipid levels were elevated by 0.90% (95% CI: −8.41–10.21%) for triglycerides (*P* = 0.849), −0.47% (−4.32–3.39%) for TC (*P* = 0.813), 0.67% (−4.85–6.20%) for HDL-C (*P* = 0.811), −2.67% (−8.72–3.39%) for LDL-C (*P* = 0.388), 0.47% (−4.85–5.79%) for non-HDL-C (*P* = 0.861), −1.53% (−7.70–4.63%) for TC/HDL-C ratio (*P* = 0.626), and −3.20% (−10.89–4.49%) for LDL-C/HDL-C ratio (*P* = 0.415), when comparing the TT genotype with the TT genotype carriers.

**Table 2:** Mean % difference (95% CI) in 3-year changes in lipid levels according to quartiles of plasma selenium concentrations^a^.

**Mean % (95% CI) difference in 3-year changes in lipid levels**	**Quartiles of plasma selenium concentrations (μg/L)**				***P-*trend**
	**Q1, <81.56**	**Q2, 81.57–91.68**	**Q3, 91.69–104.90**	**Q4, ≥104.91**	
Triglycerides					
Model 1	0.00 (referent)	1.57 (−5.25, 8.39)	1.78 (−5.00, 8.55)	6.87 (0.10, 13.64)*	0.056
Model 2	0.00 (referent)	2.85 (−3.87, 9.57)	2.73 (−3.93, 9.40)	7.42 (0.75, 14.09)*	**0.040**
Model 3	0.00 (referent)	2.90 (−3.82, 9.62)	3.13 (−3.55, 9.81)	8.25 (1.54, 14.96)*	**0.021**
TC					
Model 1	0.00 (referent)	2.70 (−0.14, 5.53)	4.08 (1.26, 6.91)*	5.40 (2.58, 8.22)*	**<0.001**
Model 2	0.00 (referent)	3.31 (0.56, 6.05)*	4.28 (1.55, 7.02)*	5.87 (3.14, 8.61)*	**<0.001**
Model 3	0.00 (referent)	3.23 (0.48, 5.98)*	4.26 (1.52, 7.00)*	5.88 (3.13, 8.63)*	**<0.001**
HDL-C					
Model 1	0.00 (referent)	1.09 (−3.10, 5.28)	−1.18 (−5.36, 3.00)	0.17 (−4.00, 4.33)	0.797
Model 2	0.00 (referent)	2.21 (−1.74, 6.16)	−0.95 (−4.88, 2.98)	0.97 (−2.96, 4.89)	0.970
Model 3	0.00 (referent)	2.14 (−1.80, 6.08)	−1.30 (−5.22, 2.62)	0.40 (−3.52, 4.32)	0.729
LDL-C					
Model 1	0.00 (referent)	3.58 (−0.76, 7.92)	4.89 (0.56, 9.21)*	6.65 (2.34, 10.96)*	**0.002**
Model 2	0.00 (referent)	4.27 (−0.03, 8.57)	5.30 (1.02, 9.59)*	7.27 (2.99, 11.55)*	**0.001**
Model 3	0.00 (referent)	4.27 (−0.04, 8.58)	5.39 (1.10, 9.69)*	7.37 (3.07, 11.67)*	**<0.001**
Non-HDL-C					
Model 1	0.00 (referent)	3.20 (−0.65, 7.04)	4.77 (0.94, 8.61)*	5.77 (1.94, 9.59)*	**0.002**
Model 2	0.00 (referent)	3.99 (0.22, 7.76)*	5.21 (1.45, 8.97)*	6.27 (2.51, 10.02)*	**0.001**
Model 3	0.00 (referent)	3.96 (0.18, 7.74)*	5.30 (1.53, 9.07)*	6.44 (2.66, 10.21)*	**<0.001**
TC/HDL-C ratio					
Model 1	0.00 (referent)	1.68 (−2.76, 6.12)	5.00 (0.59, 9.41)*	4.60 (0.20, 9.00)*	**0.016**
Model 2	0.00 (referent)	1.29 (−3.11, 5.69)	4.90 (0.53, 9.27)*	4.31 (−0.05, 8.68)	**0.019**
Model 3	0.00 (referent)	1.33 (−3.07, 5.72)	5.32 (0.95, 9.69)*	4.99 (0.62, 9.36)*	**0.007**
LDL-C/HDL-C ratio					
Model 1	0.00 (referent)	2.73 (−2.81, 8.28)	6.62 (1.11, 12.13)*	6.71 (1.22, 12.21)*	**0.007**
Model 2	0.00 (referent)	2.05 (−3.43, 7.53)	6.38 (0.93, 11.82)	6.35 (0.91, 11.79)*	**0.008**
Model 3	0.00 (referent)	2.16 (−3.32, 7.65)	6.85 (1.41, 12.30)*	7.00 (1.55, 12.46)*	**0.004**

*^a^Model 1, adjusted for respective baseline lipid (continuous); Model 2, further adjusted for sex (men/women), age (continuous), and BMI (continuous); Model 3, further adjusted for current smoking status (yes/no), current drinking status (yes/no), physical activity (yes/no), and educational level (none or elementary school/middle school/high school or beyond). BMI, body mass index; HDL-C, high-density lipoprotein cholesterol; LDL-C, low-density lipoprotein cholesterol; Non-HDL-C, non-high-density lipoprotein cholesterol; TC, total cholesterol.*

*P < 0.05. Bold values indicates P < 0.05.

**Table 3:** Mean % difference (95% CI) in 3-year changes in lipid levels according to rs7579 genotypes^a^.

**Mean % (95% CI) difference in 3-year changes in lipid levels**	**rs7579 genotypes**			***P*-trend**
	**CC**	**CT**	**TT**	
Triglycerides				
Model 1	0.00 (referent)	−2.58 (−7.61, 2.45)	2.90 (−6.55, 12.35)	0.835
Model 2	0.00 (referent)	−2.92 (−7.87, 2.03)	0.99 (−8.32, 10.30)	0.573
Model 3	0.00 (referent)	−3.13 (−8.10, 1.83)	0.90 (−8.41, 10.21)	0.532
TC				
Model 1	0.00 (referent)	−0.99 (−3.11, 1.12)	0.46 (−3.52, 4.43)	0.683
Model 2	0.00 (referent)	−1.33 (−3.38, 0.72)	−0.41 (−4.26, 3.45)	0.360
Model 3	0.00 (referent)	−1.23 (−3.28, 0.83)	−0.47 (−4.32, 3.39)	0.383
HDL-C				
Model 1	0.00 (referent)	1.86 (−1.25, 4.97)	2.25 (−3.63, 8.12)	0.223
Model 2	0.00 (referent)	1.02 (−1.91, 3.95)	0.78 (−4.76, 6.33)	0.547
Model 3	0.00 (referent)	1.36 (−1.57, 4.29)	0.67 (−4.85, 6.20)	0.473
LDL-C				
Model 1	0.00 (referent)	0.81 (−2.42, 4.04)	−1.71 (−7.81, 4.39)	0.948
Model 2	0.00 (referent)	0.49 (−2.70, 3.69)	−2.68 (−8.72, 3.36)	0.691
Model 3	0.00 (referent)	0.53 (−2.68, 3.74)	−2.67 (−8.72, 3.39)	0.704
Non-HDL-C				
Model 1	0.00 (referent)	−1.60 (−4.45, 1.26)	1.62 (−3.80, 7.03)	0.779
Model 2	0.00 (referent)	−1.97 (−4.77, 0.83)	0.51 (−4.80, 5.82)	0.470
Model 3	0.00 (referent)	−1.95 (−4.76, 0.86)	0.47 (−4.85, 5.79)	0.473
TC/HDL-C ratio				
Model 1	0.00 (referent)	−3.03 (−6.33, 0.26)	−1.88 (−8.10, 4.35)	0.134
Model 2	0.00 (referent)	−2.61 (−5.88, 0.65)	−1.56 (−7.74, 4.61)	0.196
Model 3	0.00 (referent)	−2.89 (−6.16, 0.38)	−1.53 (−7.70, 4.63)	0.165
LDL-C/HDL-C ratio				
Model 1	0.00 (referent)	−1.12 (−5.23, 3.00)	−3.99 (−11.77, 3.79)	0.318
Model 2	0.00 (referent)	−0.54 (−4.61, 3.52)	−3.29 (−10.99, 4.40)	0.471
Model 3	0.00 (referent)	−0.84 (−4.92, 3.24)	−3.20 (−10.89, 4.49)	0.427

*^a^Model 1, adjusted for respective baseline lipid (continuous); Model 2, further adjusted for sex (men/women), age (continuous), and BMI (continuous); Model 3, further adjusted for current smoking status (yes/no), current drinking status (yes/no), physical activity (yes/no), and educational level (none or elementary school/middle school/high school or beyond). BMI, body mass index; HDL-C, high-density lipoprotein cholesterol; LDL-C, low-density lipoprotein cholesterol; Non-HDL-C, non-high-density lipoprotein cholesterol; TC, total cholesterol*.

**Figure 1:**
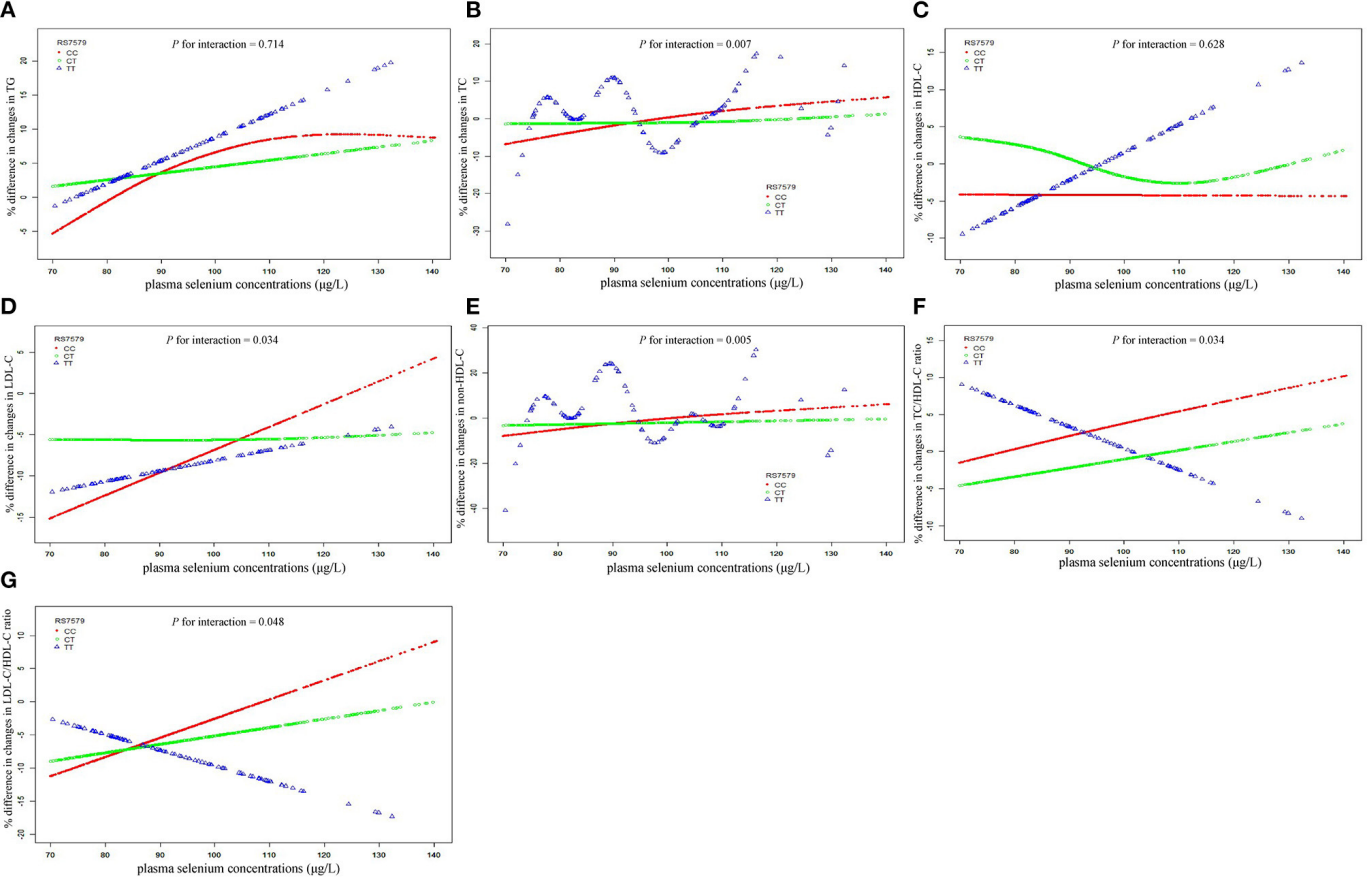
Associations of plasma selenium concentrations with 3-year changes in lipids stratified by rs7579 genotypes. X bars represent plasma selenium concentrations. Y bars represent mean % difference in 3-year changes in triglycerides for **(A)**, TC for **(B)**, HDL-C for **(C)**, LDL-C for **(D)**, non-HDL-C for **(E)**, TC/HDL-C ratio for **(F)**, and LDL-C/HDL-C ratio for **(G)**. Multivariable analyses were adjusted for sex (men/women), age (continuous), BMI (continuous), current smoking status (yes/no), current drinking status (yes/no), physical activity (yes/no), educational level (none or elementary school/middle school/high school or beyond), and respective baseline lipid (continuous). BMI, body mass index; HDL-C, high-density lipoprotein cholesterol; LDL-C, low-density lipoprotein cholesterol; Non-HDL-C, non-high-density lipoprotein cholesterol; TC, total cholesterol.

Analyses of plasma selenium with 3-year changes in lipid levels were stratified by rs7579 genotypes (Figure 1). In stratified analyses, positive associations between plasma selenium concentrations and 3-year changes in triglycerides, TC, LDL-C, non-HDL-C, TC/HDL-C ratio, and LDL-C/HDL-C ratio were observed among CC genotype carriers, but negative associations between plasma selenium and TC/HDL-C ratio, and LDL-C/HDL-C ratio were observed among TT genotype carriers. Among rs7579 CC genotype carriers, comparing the highest to the lowest quartiles of plasma selenium concentrations, 3-year changes in lipid levels were elevated by 11.54% (2.29–20.79%) for triglycerides (*P* = 0.015), 9.39% (5.53–13.24%) for TC (*P* < 0.001), −0.41% (−5.94–5.11%) for HDL-C (*P* = 0.884), 11.66% (5.72–17.61%) for LDL-C (*P* = 0.0001), 10.91% (5.64–16.18%) for non-HDL-C (*P* < 0.001), 9.44% (3.36–15.51%) for TC/HDL-C ratio (*P* = 0.002), and 12.35% (5.19–19.51%) for LDL-C/HDL-C ratio (*P* = 0.0007). Among TT genotype carriers, comparing the highest to the lowest quartiles of plasma selenium concentrations, 3-year changes in lipid levels were elevated by 26.45% (−3.43–56.33%) for triglycerides (*P* = 0.082), 6.23% (−4.65–17.10%) for TC (*P* = 0.259), 15.41% (−6.15–36.97%) for HDL-C (*P* = 0.159), 8.56% (−14.93–32.06%) for LDL-C (*P* = 0.471), 4.06% (−11.20–19.31%) for non-HDL-C (*P* = 0.599), −5.45% (−32.65–21.76%) for TC/HDL-C ratio (*P* = 0.692), and −3.88% (−41.99–34.24%) for LDL-C/HDL-C ratio (*P* = 0.840). The interactions between plasma selenium and rs7579 polymorphism on 3-year changes in TC (*P* for interaction = 0.007), LDL-C (*P* for interaction = 0.034), non-HDL-C (*P* for interaction = 0.005), TC/HDL-C ratio (*P* for interaction = 0.034), and LDL-C/HDL-C ratio (*P* for interaction = 0.048) were statistically significant.

## Discussion

In this study, plasma selenium was positively associated with 3-year changes in triglycerides, TC, LDL-C, non-HDL-C, TC/HDL-C ratio, and LDL-C/HDL-C ratio. Moreover, the positive associations between plasma selenium and 3-year changes in lipid levels were only significant in rs7579 CC genotype carriers, while non-significant associations were observed in T allele carriers.

The selenium status among people varies by regions and mainly depends on its dietary intake (20, 21). The intakes are high in America, Japan, and Canada, and much lower in the majority of Eastern Europe. China has both high-selenium and low-selenium areas (20). Although the optimal selenium status remains controversial, findings from some studies suggested the plasma selenium concentration around 90 μg/L to be the optimal value, which maximizes the plasma SELENOP level (20). The median concentration of plasma selenium was 91.68 μg/L in our participants, which was considered as selenium-sufficient.

Our findings of positive associations between plasma selenium and changes in triglycerides, TC, LDL-C, non-HDL-C, TC/HDL-C ratio, and LDL-C/HDL-C ratio are consistent with those from cross-sectional studies conducted among subjects with a wide-range of selenium status, which also demonstrated strong, positive associations between selenium status and lipid levels (3, 4, 21–24). For example, in the third National Health and Nutrition Examination Survey (NHANES III), which included a representative American population (median blood selenium 199.88 μg/L), increased serum selenium was significantly associated with higher lipid levels (21). Also, another cross-sectional study conducted among a selenium-excess Chinese population (mean serum selenium 120 μg/L) revealed significant positive associations of plasma selenium with triglycerides, TC, and LDL-C (3). In addition, several cross-sectional studies conducted among selenium-deficient individuals (mean serum selenium 79.5 μg/L) also revealed that higher plasma selenium was associated with adverse lipid levels (7, 25). Limited longitudinal studies have explored relationships between baseline blood selenium and changes in lipid levels, and findings from them indicated non-significant or negative associations between selenium status and changes in lipid levels (6, 8). It seems likely that there are some other unmeasured factors might confound the longitudinal association between selenium status and lipid levels. Additionally, in view of genetic factors account for nearly 30% of lipids, such contradictory findings might be partly due to difference in genetic background of participants between studies. More longitudinal studies examining associations of selenium status with changes in lipid levels are warranted to further verify our findings.

Although exact mechanisms underlying the adverse effects of increased selenium on lipid changes among selenium-sufficient or selenium-excess subjects are not clearly understood, several potential mechanisms might be involved, including endoplasmic reticulum stress, mitochondrial dysfunction, and changed miRNA expression. Firstly, excess selenium has been suggested to induce endoplasmic reticulum stress (26). Under endoplasmic reticulum stress, the expression of several key factors in the pathway of *de novo* lipogenesis was upregulated in the liver, including the sterol regulatory element binding protein 1c (SREBP1c) and the fatty acid synthase (FASN) (27). Secondly, high selenium might be involved in abnormal lipid metabolism through disturbing mitochondrial function. Romero et al. revealed that high selenium exposures could induce mitochondrial dysfunction by increasing the generation of reactive oxygen species (28). Mitochondrial dysfunction could disturb lipid metabolism through several mechanisms, including leading to endoplasmic reticulum stress, impairing autophagy, and then increasing lipogenesis (29, 30). And lastly, Guo et al. found blood selenium was positively associated with the expression of miR-122-5p (31), which has been implicated as a key regulator of cholesterol and fatty-acid metabolism in the liver (32). Nevertheless, more mechanistic studies are needed to further illustrate potential mechanisms through which high selenium plays a role in adverse changes in lipid levels.

Our study also revealed significant interactions between plasma selenium and rs7579 polymorphism on changes in lipid levels, which helped to explain the inconsistent findings between studies and suggested that such inconsistency might be partly due to the selenium × rs7579 polymorphism interactions. We showed that the impact of baseline selenium on changes in lipid levels depended on genotype at rs7579. Notably, the adverse effect of high selenium on changes in lipid levels was only significant among rs7579 CC genotype carriers, but non-significant among rs7579 T allele carriers. Although such an interaction has not been reported before, there are studies that corroborate our findings. Findings from an intervention trial suggested that the changes in cholesterol levels in response to Brazil nut supplementation were greater among rs7579 CC genotype than T allele carriers (18), which is in line with the currently observed differential associations of plasma selenium with lipid changes across rs7579 genotypes. Furthermore, it has been suggested that both the mRNA expression and plasma levels of SELENOP were significantly lower among rs7579 CC than T allele carriers (16, 17), and decreased plasma SELENOP was related with increased risks of several dyslipidemia-related diseases, including metabolic syndrome, cerebrovascular events, and cardiovascular disease (33–35). Our study also revealed non-significant associations between rs7579 polymorphism and changes in lipid levels, which is consistent with those from previous studies.

Our study has several strengths. First, to our knowledge, this is the first population-study systematically exploring the interactions between plasma selenium and rs7579 polymorphism with changes in lipid levels. Second, the prospective design allowed us to clarify the temporal sequence of the relationships between selenium and lipid levels. Third, we assess selenium status using plasma selenium concentration instead of dietary intake of selenium to avoid possible bias through dietary survey.

Several limitations should also be acknowledged. First, plasma selenium concentrations were measured only once, and measurement errors and exposure misclassification were inevitable. However, such errors were likely to be non-differential and thus would be more likely to induce the attenuation of the observed associations. Second, we only measured total plasma selenium levels rather than different chemical forms of selenium. Given that different chemical forms of selenium might have different properties and effects on human health, more detailed analyses of different chemical forms of selenium are needed to better understand the relationships between selenium and lipid changes. Also, we did not have measurement of plasma SELENOP levels, which precluded us from exploring the associations of rs7579 polymorphism as well as lipid changes with plasma SELENOP levels. Third, our study was conducted among a selenium-appropriate population. Thus, the generalizability of our findings to people with different selenium statuses remains unclear, and further studies are needed to determine the generalizability of our findings. Fourth, the study population was limited to a Chinese population, which also limits the generalizability of our findings. Finally, no information on health status (e.g., obese, diabetic) was collected in our population, which should be taken into account in future study. In addition, although multiple potential confounders have been adjusted, we could not rule out the possibility of residual confounding by other unknown or unmeasured factors.

In conclusion, our findings demonstrated that, in a selenium-appropriate population, plasma selenium was associated with lipid changes differentially across rs7579 genotypes, and higher selenium seemed to be associated with adverse changes in lipid levels among rs7579 CC genotype carriers, but not among T allele carriers. These findings warn the widespread use of selenium-fortified foods or selenium supplements, especially among selected study populations, such as rs7579 CC genotype carriers.

## Data Availability Statement

The raw data supporting the conclusions of this article will be made available by the authors, without undue reservation.

## Ethics Statement

The studies involving human participants were reviewed and approved by the Ethics and Human Subject Committee of Tongji Medical College. The patients/participants provided their written informed consent to participate in this study.

## Author Contributions

LZ contributed to the statistical analysis, interpretation of the results, and wrote the manuscript. LZ, XLia, MX, JY, and YH contributed to the acquisition of data and researched the data. XLi, ZS, and LC contributed to the study design. YZ contributed to the discussion of the project. LZ, XLia, MX, JY, YH, XLi, ZS, LC, YZ, CL, and LL reviewed and edited the manuscript. LL and CL are the guarantors of this work and as such, have full access to all the data in the study and take responsibility for the integrity of the data and the accuracy of the data analysis. All authors contributed to the article and approved the submitted version.

## Funding

This work was supported by the Major International (Regional) Joint Research Project (NSFC 81820108027), the National Key Research and Development Program of China (2020YFC2006300), the National Natural Science Foundation of China (81803226), and the Angel Nutrition Research Fund (AF2019003). The funding sources had no role in the design and conduct of the study, collection, management, analysis, and interpretation of the data, preparation, review, or approval of the manuscript, and decision to submit the manuscript for publication.

## Conflict of Interest

The authors declare that the research was conducted in the absence of any commercial or financial relationships that could be construed as a potential conflict of interest.

## Publisher's Note

All claims expressed in this article are solely those of the authors and do not necessarily represent those of their affiliated organizations, or those of the publisher, the editors and the reviewers. Any product that may be evaluated in this article, or claim that may be made by its manufacturer, is not guaranteed or endorsed by the publisher.

## Abbreviations

BMI: body mass index
FASN: fatty acid synthase
HDL-C: high-density lipoprotein cholesterol
LDL-C: low-density lipoprotein cholesterol
NHANES III: the third National Health and Nutrition Examination Survey
SELENOP: selenoprotein P
SREBP1c: sterol regulatory element binding protein 1c
TC: total cholesterol
TJEZ: Tongji-Ezhou.
